# Inactivated *Vibrio cholerae* Strains That Express TcpA via the *toxT*-139F Allele Induce Antibody Responses against TcpA

**DOI:** 10.4014/jmb.2209.09001

**Published:** 2022-10-12

**Authors:** Eun Jin Kim, Jonghyun Bae, Young-Jun Ju, Do-Bin Ju, Donghyun Lee, Seonghyeon Son, Hunseok Choi, Thandavarayan Ramamurthy, Cheol-Heui Yun, Dong Wook Kim

**Affiliations:** 1 Department of Pharmacy, College of Pharmacy, Hanyang University, Ansan 15588, Republic of Korea; 2 Institute of Pharmacological Research, Hanyang University, Ansan 15588, Republic of Korea; 3 Department of Agricultural Biotechnology, and Research Institute of Agriculture and Life Sciences, Seoul National University, Seoul 08826, Republic of Korea; 4 ICMR-National Institute of Cholera and Enteric Diseases, Kolkata 700010, India

**Keywords:** Cholera, toxin coregulated pilus (TCP), TCP major subunit A (TcpA), *Vibrio cholerae*, vaccine

## Abstract

Cholera remains a major global public health problem, for which oral cholera vaccines (OCVs) being a valuable strategy. Patients, who have recovered from cholera, develop antibody responses against LPS, cholera toxin (CT), toxin-coregulated pilus (TCP) major subunit A (TcpA) and other antigens; thus, these responses are potentially important contributors to immunity against *Vibrio cholerae* infection. However, assessments of the efficacy of current OCVs, especially inactivated OCVs, have focused primarily on O-antigen-specific antibody responses, suggesting that more sophisticated strategies are required for inactivated OCVs to induce immune responses against TCP, CT, and other antigens. Previously, we have shown that the *toxT*-139F allele enables *V. cholerae* strains to produce CT and TCP under simple laboratory culture conditions. Thus, we hypothesized that *V. cholerae* strains that express TCP via the *toxT*-139F allele induce TCP-specific antibody responses. As anticipated, *V. cholerae* strains that expressed TCP through the *toxT*-139F allele elicited antibody responses against TCP when the inactivated bacteria were delivered via a mouse model. We have further developed TCP-expressing *V. cholerae* strains that have been used in inactivated OCVs and shown that they effect an antibody response against TcpA in vivo, suggesting that *V. cholerae* strains with the *toxT*-139F allele are excellent candidates for cholera vaccines.

## Introduction

Cholera is a potentially fatal diarrheal disease that is caused by the gram-negative bacteria *Vibrio cholerae* [1, 2]. Of the more than 200 serogroups that are based on the O-antigen, O1 and O139 produce cholera toxin (CT), which is an enterotoxin considered the primary virulence factor to evoke disease symptoms. O1 serogroup strains are divided into the Ogawa, Inaba, and Hikojima serotypes, depending on the presence and degree of terminal methyl groups on the O-antigen [3, 4]. In addition, O1 strains are classified into 2 biotypes – classical and El Tor –based on their microbiological characteristics [5, 6].

Toxin coregulated pilus (TCP) is another major virulence factor in *V. cholerae* infections [3]. TCP is pivotal in the bacterial colonization of cells in the small intestine in humans [7-9]. TCP also acts as the receptor for the cholera toxin phage (CTXΦ) that provides *V. cholerae* with cholera toxin genes [10].

*tcpA* is composed of 675 nucleotides that are translated into the 224-amino-acid TcpA pilin (20.3 kDa) and TCP comprises a homopolymer of TcpA. The N-terminal 25 amino acids of TcpA are removed during polymerization to a pilus [11]. TcpAs of the *V. cholerae* O1 serogroup El Tor (TcpA-ET) and classical (TcpA-cla) biotype strains share 85% amino acid similarity, whereas the O139 serogroup strains contain identical TcpAs as those of O1 El Tor biotype strains [12]. A new variant of TcpA (Haitian type TcpA), which differs by a single amino acid (position 89, Asn to Ser) from the El Tor biotype TcpA, was identified in *V. cholerae* strains that caused the cholera outbreak in Haiti in 2010 [13]. Furthermore, *V. cholerae* strains that contain the Haitian type TcpA have been prevalent in recent global cholera outbreaks [14, 15].

Patients who have recovered from cholera have protective immunity against subsequent *V. cholerae* infections [16, 17]. Further, analyses of immune responses against *V. cholerae* have shown that antibody responses against O-antigens, CT, TCP, and other protein antigens are important in conferring immunity against *V. cholerae* [18-20].

Two types of oral cholera vaccines (OCVs) —inactivated and live attenuated vaccines—are available [16, 21]. Inactivated vaccines contain killed whole-cell O1 serogroup strains plus an O139 serogroup strain - *i.e.*, Shanchol and Euvichol—or comprise a mixture of inactivated O1 serogroup strains and recombinant cholera toxin B (CTB) subunit - *i.e.*, Dukoral [22]. Notably, the *V. cholerae* cells in inactivated vaccines are cultured under conditions that do not allow the production of TCP/CT; thus, immune responses against TCP/CT are not anticipated after the vaccination with inactivated vaccines.

A live-attenuated vaccine (Vaxchora), approved by the US Food and Drug Administration [16, 23], was developed by deleting most of the toxin A gene (*ctxA*) in an O1 serogroup classical biotype Inaba serotype strain, 569B, and was shown to induce immune responses against O-antigens and CTB [24-26]. This vaccine was expected to mimic the natural infection by *V. cholerae* and to induce immune responses against TCP, but instead, a priming effect for a subsequent response to TcpA at a 90-day challenge has been reported [27].

Nonetheless, TcpA and synthetic internal peptides of TcpA have been shown to be immunogenic and induce protective immunity in animal models [28-30]. However, multiple exposures to TcpA are required to induce anti-TCP responses in endemic settings, suggesting that TCP is not a strong immunogen in natural infections or vaccination. Thus, it is most likely that the efficient delivery of TCP is essential for whole-cell vaccines to elicit anti-TCP responses and to improve immune responses.

The expression of CT and TCP during *V. cholerae* infection is controlled by ToxT, which is regulated by ToxR/ToxS and TcpP/TcpH in the intestinal environment [30, 31]. Under laboratory conditions, the expression of CT and TCP in *V. cholerae* O1 serogroup classical biotype strains requires a simple culture method (*i.e.*, agglutinating conditions, bacterial culture at 30°C in LB medium, and pH adjusted to pH 6.5 with 50 mM Tris). Various culture methods have been developed to express CT and TCP in El Tor biotype strains [32-35], but the induction of immune responses against TcpA by such strains has yet to be examined in detail.

Recently, we have reported that the *toxT*-139F allele facilitates the production of CT in El Tor and classical biotype strains [36-38]. In the current study, *V. cholerae* strains—O395-*toxT*-139F, a derivative of the classical biotype strain O395, and IB5230-*toxT*-139F, a derivative of a Wave 3 El Tor biotype strain—that produce CT *via* the *toxT*-139F allele were examined for their ability to stably express TCP and induce antibody responses against TCP in an animal model. We then expressed TCP in the *V. cholerae* strains that are in OCVs to evaluate this approach for practical applications. As a result, robust antibody responses against TCP were induced, implying that the *toxT*-139F allele can be incorporated into OCVs to improve their efficacy.

## Materials and Methods

### Bacterial Strains

The bacterial strains that are used in this study are listed in Table 1.

### Antibodies

Rabbit anti-TcpA against classical TcpA was a generous gift from Dr. W. F. Wade, Dartmouth University, Hanover, NH, USA [30]. Goat anti-rabbit IgG and goat anti-mouse IgG secondary antibodies were purchased from LI-Cor Biosciences (USA).

### Bacterial Culture and TcpA Expression

TcpA expression in *V. cholerae* strains was examined in various culture media (LB, LB Tris-Cl pH 6.5, PBS-buffered LB, and AKI media) at 30°C or 37°C as described [37]. The optimal culture conditions for expression of TcpA for the bacterial strains were determined as follows; *E. coli* DH5α, *V. cholerae* O395, YJB001 (O395-*toxT*-139F), EJK003 (Cairo48-*toxT*-139F), JHB001 (Cairo48-*toxT*-139F-*tcpA*-ET), EJK005 (Phil6973-*toxT*-139F), EJK006 (4260B-*toxT*-139F), 569B, and EJK007 (569B-*toxT*-139F) have been cultured in LB media at 30°C. *V. cholerae* strains EJK001 (IB5230-ch1-kan), EJK002 (IB5230-ch1-kan-*toxT*-139F), and EJK004 (Cairo50-*toxT*-139F) were cultured in AKI media at 30°C without 4 h of static incubation, distinguishing this protocol from AKI culture conditions (4 h of static incubation, followed by 16 h of vigorous shaking at 37°C in AKI media), which has been applied to induce CT in El Tor biotype strains [35].

### Allelic Exchange of *ctxA*B with a Kanamycin Cassette

A kanamycin-resistance gene cassette was PCR-amplified from the pET-24d(+) vector (Novagen, Germany) using the primer set Kan-BamHI-F: CCG GGA TCC CCG GCA GAT TAC GCG CAG AAA and Kan-PstI-R: GGC CTG CAG GGC TTA GAA AAA CTC ATC GAG and inserted between the BamHI/PstI sites of pUC18-zot to generate pUC18-Kan. A 521-bp DNA fragment (from the nucleotide 778 of *zot* to the end of the intergenic sequence between *zot* and *ctxA*) was amplified by PCR from genomic DNA of strain V212-1using the primer pair Zot-EcoRI-F: CCG GAA TTC GCG TCA GAG CAA TCC GAG CCT and Zot-BamHI-R: GCG GGA TCC CCG ATA ATG CTC CCT TTG TTT. The amplified DNA fragment was inserted between the EcoRI/BamHI sites of pUC18 to generate pUC18-zot-Kan (Fig. S1). An 832-bp DNA fragment that encompassed the C-terminal 209 bp of *ctxB* and the entire non-coding sequence between the CTX prophage and RS1 element of strain V212-1 was PCR-amplified from V212-1 genomic DNA using the primer pair *ctxB*-ig-PstI-F: GGC CTG CAG GGC GAG AGA TGG CTA TCA TTA and *ctxB*-ig-HindIII-R: CCG AAG CTT GCG CAT CTT AAA TCA TGG TGC. The PCR-amplicon was then inserted into the PstI/HindIII sites of pUC18-zotKan to generate pUC18-zotKan*ctxB*.

The entire fragment that was inserted into pUC18-zotKan*ctxB* was PCR-amplified using the primer pair Zot-XbaI F: CCG TCT AGA GCG TCA GAG CAA TCC GAG CCT and SacI-ch1R: CCG GAG CTC GCG CAT CTT AAA TCA TGG TGC and subcloned in the XbaI/SacI site of the pCVD442 suicide plasmid to generate pCVD-zotKan*ctxB* (Fig. S1). The *E. coli* strain SM10 was transformed with pCVD-zot-Kan-*ctxB* to replace the entire *ctxA* gene and the first 166 bp of *ctxB* of strain IB5230 with a kanamycin resistance cassette by allele exchange method to generate the strain EJK001 (IB5230-ch1-kan).

### Allelic Exchange of *toxT*-139Y with *toxT*-139F in *V. cholerae* Strains

An 843-bp DNA fragment that spanned the 50 nucleotides upstream of the translation start codon to nucleotide 793 of *toxT* was PCR-amplified from strain MG116025, which contains the *toxT*-139F allele, using the primers *toxT*-XbaI-F: CCG GCC TCT AGA TAC GTG GAT GGC TCT CTG CG and *toxT*-SacI-R: CCG GCC GAG CTC CAC TTG GTG CTA CAT TCA. The PCR-amplified fragment was inserted into the pCVD442 suicide plasmid to construct pCVD442-*toxT*-139F. pCVD442-*toxT*-139F was conjugally transferred to strains O395, N16961, T19479, EJK001 (IB5230-ch1-kan), Cairo48, Cairo50, Phil6973, 4260B, and 569B to replace their *toxT*-139Y allele with *toxT*-139F. The replacement of the *toxT* allele in each strain was confirmed by sequencing [36, 37].

### Purification of His-Tagged TcpA-El Tor and TcpA-cla

Full-length *tcpA*-ET (El Tor) was PCR-amplified from the *V. cholerae* O1 serogroup strain N16961 using the primer pair TcpA-NcoI-F: GGG CCA TGG AAT TAT TAA AAC AGC TTT TTA AG and TcpAET-XhoI-R: GGG CTC GAG ACT GTT ACC AAA AGC TAC TG. Similarly, full-length *tcpA*-cla was PCR-amplified from the *V. cholerae* O1 serogroup strain O395 using the primer pair TcpA-NcoI-F and TcpACla-XhoI-R: GGG CTC GAG GCT GTT ACC AAA TGC AAC G.

PCR-amplified *tcpA*-ET and *tcpA*-cla were then subcloned between the NcoI and XhoI sites of pET24-(d)(Novagen, Merck) to generate pET24-TcpAET and pET24-TcpACla, respectively. The recombinant plasmids were used to transform *E. coli* BL21 (DE3). His-tagged TcpA-ET and TcpA-cla were purified using TALON metal affinity resin (Takara Bio Inc., Japan) according to the manufacturer’s instructions (Fig. S2). The molecular weight of His-tagged TcpA was approximately 24 kDa, and that of cellular TcpA was roughly 20.3 kDa. Purification of His-tagged TcpA-ET and TcpA-cla was confirmed by western blot using anti-TcpA [30].

### Replacement of *tcpA*-cla with *tcpA*-ET in the Classical Biotype Strain Cairo48

A 2,565 bp DNA fragment that spanned from nucleotide 355 of tcpP to nucleotide 562 of tcpB was amplified using the primer pair tcpP-XbaIF: GGG TCT AGA GTT GAT GAA GCT GAC TGT AGT C and *tcpA*-SacIR-1: CCC GAG CTC CGA TTC GAT AGT CTT GTG CGC from genomic DNA of the El Tor biotype strain N16961. This fragment was digested with XbaI and SacI and inserted into the XbaI and SacI site of the pCVD442 suicide plasmid to generate pCVD-*tcpA*-ET. pCVD-*tcpA*-ET was conjugally transferred to strain EJK003 (Cairo48-*toxT*-139F) to replace the *tcpA*-cla allele with *tcpA*-ET, resulting in strain JHB001 (Cairo48-*toxT*-139F-*tcpA*-ET), as confirmed by sequencing.

### SDS-PAGE and Western Blotting

Bacterial cells (approximately 10^7^ cells/well) and purified TcpA proteins (approximately 2 μg/well) were analyzed using 15% SDS-PAGE. Proteins were visualized by Coomassie brilliant blue staining. For western blotting, proteins separated by SDS-PAGE were transferred onto a nitrocellulose membrane. Membrane was blocked for one hour at room temperature in TBS blocking buffer. Primary antibodies (anti-TcpA was diluted 1:10,000 and the mouse antisera were diluted 1:5,000) were added to the membrane and incubated overnight at 4°C. Membrane was then washed with TBST and secondary antibody [goat anti-rabbit IgG (diluted 1:20,000) or goat anti-mouse IgG (diluted 1:5,000)] was added. Western blot image was analyzed by Odyssey CLx imaging system (LI-COR Biosciences, USA). The western blot band intensities representing TcpA were quantified using ImageJ gel analysis program and LI-COR Odyssey software.

### Mice and Ethics Statement

Female BALB/c mice aged at 8-10 weeks, purchased from Jackson Laboratory, were maintained under specific pathogen-free conditions with a 12-h light/dark cycle at 25°C and 40% humidity and free access to food and water. Unless described otherwise, all experiments using mice were performed according to protocols that were approved by the Institutional Animal Care and Use Committee of Seoul National University (SNU-210216-1-1).

### Immunization of Mice with Inactivated Bacteria

The bacterial cells, cultured as aforementioned, were harvested and resuspended in PBS (5 × 10^9^ cells/ml) and heat-inactivated at 65°C for 90 min in a water bath. The viability of the heat-killed bacteria was determined by inoculating LB agar plates and confirmed, based on the absence of growth after overnight incubation at 37°C (data not shown).

For in vivo studies, mice were allocated into groups of 4 animals and immunized by intraperitoneal route with heat-inactivated bacteria (1 × 10^9^ CFU in 200 μl). Two sets of bacterial strains were examined: Set 1 (six groups) consisted of PBS as a negative control, *E. coli* DH5α, *V. cholerae* O395, YJB001, EJK001, and EJK002. Set 2 (9 groups) comprised PBS as a negative control, *E. coli* DH5α, Cairo48, EJK003 (Cairo48-*toxT*-139F), EJK004 (Cairo50-*toxT*-139F), Phil6973, EJK005 (Phil6973-*toxT*-139F), EJK006 (4260B-*toxT*-139F), and JHB001 (Cairo48-*toxT*-139F-*tcpA*-ET).

Mice were immunized with 1 × 10^9^ CFU in 200 μl PBS on Days 0, 14, and 28. To measure the antibody production, at least 200 μl of blood was collected and centrifuged at 13,000 ×*g* for 15 min to separate the serum from red blood cells. Serum samples were obtained 7 days after each immunization. Animal immunization experiments were performed in triplicate.

### ELISA for TcpA-Specific IgG Antibodies in Serum

Antibodies specific for TcpA in antisera against TCP-expressing *V. cholerae* strains were quantified by ELISA as previously described [37]. Briefly, ELISA plates were coated with 100 μl His-tagged TcpA-ET (5 μg/ml) or His-tagged TcpA-cla (2.5 μg/ml) per well. Serum was applied from 1:500 to 1:32,000 (2-fold serial dilutions). Following a wash step, 100 μl HRP-conjugated secondary goat anti-mouse IgG, diluted 1:5,000 was applied. Plates were read spectrophotometrically at 450 nm.

## Results

### Expression of TcpA in *V. cholerae* Strains that Contain the *toxT*-139F Allele

We have reported the production of CT through introduction of the *toxT*-139F allele in *V. cholerae* strains (the O395 classical biotype strain; the N16961 and T19479 Wave 1 El Tor biotype strains; the B33, MJ1236, and MG116025 Wave 2 El Tor biotype strains; and the IB4122 and IB5230 Wave 3 El Tor biotype strains) [37]. In the present study, TCP production and their potential to induce antibody responses in an animal model on immunization have been examined. The expression of TcpA in *V. cholerae* strains (Table 1) was evaluated by western blot using anti-TcpA [30].

**Fig. 1:**
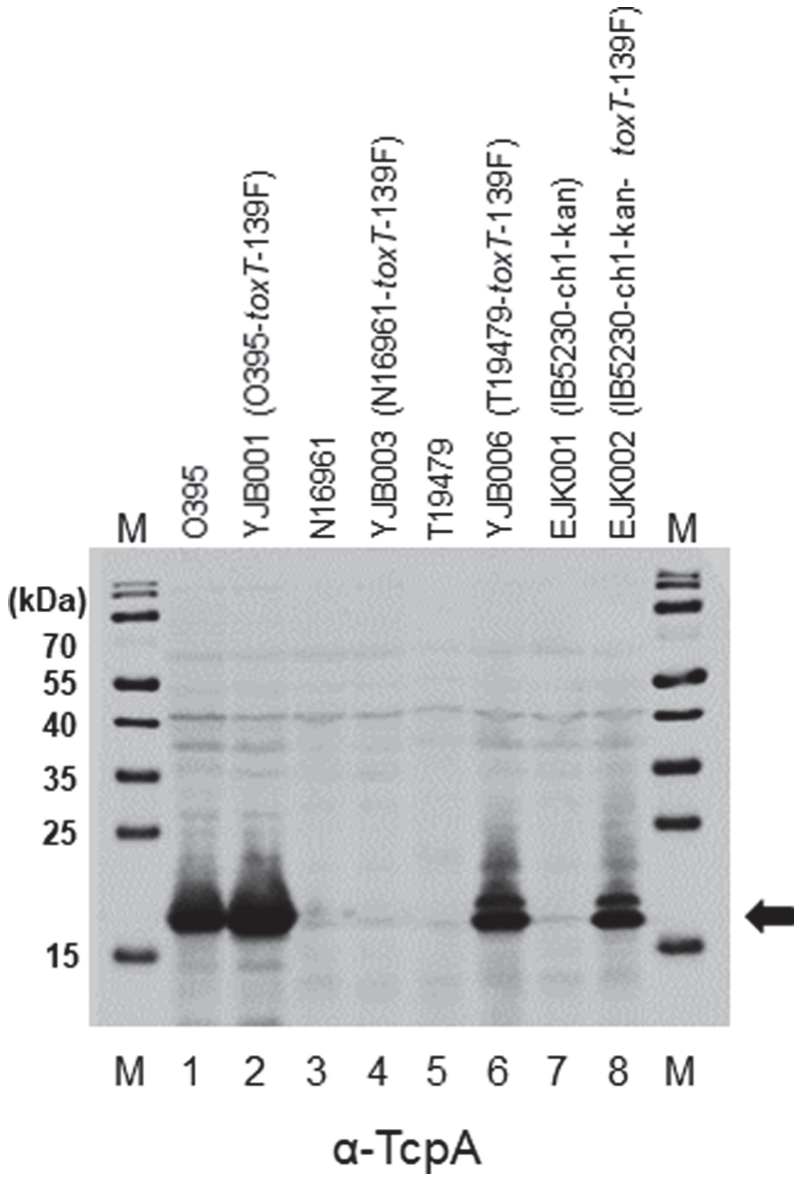
Expression of TcpA in *V. cholerae* strains. Bacterial strains were cultured at 30°C in LB media except for IB5230 and EJK002 (IB5230-ch1-kan-*toxT*-139F), which were cultured in AKI media at 37°C (without 4 h of static incubation, distinguishing this protocol from AKI culture conditions). Cellular TcpA was detected by western-blot with anti-TcpA. Approximately 1 × 10^7^ cells were analyzed. M: molecular weight marker (kDa), lane 1: O395, lane 2: YJB001 (O395-*toxT*-139F), lane 3: N16961, lane 4: YJB003 (N16961-*toxT*-139F), lane 5: T19479, lane 6: YJB006 (T19479-*toxT*-139F), lane 7: EJK001 (IB5230-ch1-kan), and lane 8: EJK002 (IB5230-ch1-kan-*toxT*-139F). Arrow indicates cellular TcpA, the molecular weight of which was approximately 21 kDa. Coomassie Brilliant Blue-stained SDS-PAGE gel of the same bacterial cells is shown in Fig. S1.

The O395 classical biotype strain contains the *toxT*-139Y allele and produces CT under simple culture conditions (in LB media at 30°C) and under agglutinating conditions [37, 38]. TcpA was also produced from O395 cells that were cultured in LB at 30°C (lane 1, Figs. 1. and S3). An isogenic derivative of O395 that contains the *toxT*-139F allele (YJB001) synthesized approximately 4-fold more CT than parent O395 cells [37], and the expression of TcpA was markedly higher in YJB001 than O395 (band intensity was 2.5-fold higher) under the same culture conditions (lane 2. Figs. 1 and S3). This phenomenon was observed in 569B but not in Cairo48 and Cairo50—all of which are classical biotype strains (described below, Fig. 4).

We evaluated the expression of TcpA in 2 Wave 1 El Tor biotype strains (N16961 and T19479) and a derivative of a Wave 3 strain IB5230 (EJK001, in which the cholera toxin gene of IB5230 has been replaced with a kanamycin resistance cassette). No TcpA was produced by the El Tor biotype strains that contained the *toxT*-139Y allele under single-phase culture conditions (lanes 3, 5, and 7, Fig. 1). Intriguingly, when the *toxT*-139Y allele was replaced with *toxT*-139F, TcpA was generated by YJB006 (T19479-*toxT*-139F) and EJK002 (IB5230-ch1-kan-*toxT*-139F), whereas no TcpA was produced by YJB003 (N16961-*toxT*-139F) (lanes 6, 8, and 4, Fig. 1). The production of TcpA by El Tor biotype strains that contain the *toxT*-139F allele in the present study coincides with that of CT [37].

Collectively, these results indicate that the *toxT*-139F allele facilitates the expression of TcpA and CT in El Tor biotype strains as well as classical biotype strains under laboratory conditions. Consequently, EJK001 (IB5230-ch1-kan), EJK002 (IB5230-ch1-kan-*toxT*-139F), O395, and YJB001 (O395-*toxT*-139F) were used in animal immunization experiments to assess antibody responses against TcpA.

### Detection of Cellular TcpA and Purified His-Tagged TcpA

Six groups of mice were immunized 3 times at 2-week intervals with approximately 1 × 10^9^ inactivated bacterial cells [*E. coli* DH5α, *V. cholerae* EJK001 (IB5230-ch1-kan), EJK002 (IB5230-ch1-kan-*toxT*-139F), O395, and YJB001 (O395-*toxT*-139F); PBS was used as a negative control]. Antisera were obtained 7 days after each immunization, and the presence of anti-TcpA in the antisera after the third immunization was determined by western blot.

**Fig. 2:**
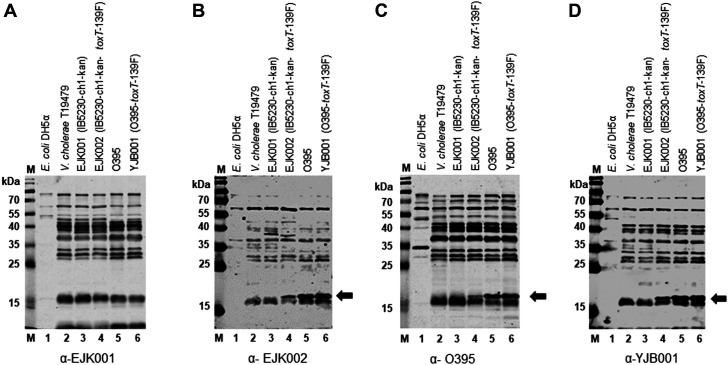
Western blot analysis of bacterial strains using mouse antisera obtained in response to immunization with inactivated bacterial strains. Western blot with antisera against: (**A**) inactivated EJK001 (IB5230-ch1-kan), (**B**) inactivated EJK002 (IB5230-ch1-kan-*toxT*-139F), (**C**) inactivated O395, and (**D**) inactivated YJB001 (O395-*toxT*-139F). Approximately 10^7^ bacterial cells were analyzed. Lane 1: *E. coli* DH5α, lane 2: *V. cholerae* T19479, lane 3: EJK001 (IB5230-ch1-kan), lane 4: EJK002, lane 5: O395, and lane 6: YJB001 (O395-*toxT*-139F). Arrow indicates cellular TcpA (approximately 21 kDa). Results of western blot analysis with mouse antisera against *E. coli*, anti-TcpA, and Coomassie Brilliant Blue staining of the same samples are shown in Fig. S2.

Mouse antiserum that was raised against inactivated EJK001 reacted with multiple *V. cholerae* proteins but not cellular TcpA (Fig. 2A). Antisera against EJK002, O395, and YJB001 reacted against cellular TcpA in *V. cholerae* strains that expressed TcpA - EJK002, O395, and YJB001 (lanes 4-6, Figs. 2B-2D). It was evident that antisera against *E. coli* failed to react with TcpA (Fig. S4).

**Fig. 3:**
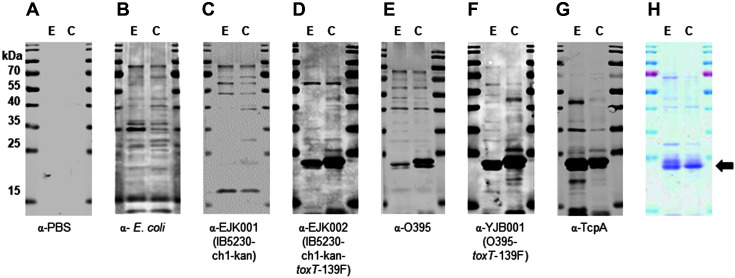
Western blot analysis of His-tagged TcpA-ET and TcpA-cla using mouse antisera in response to immunization with inactivated bacterial strains. Western blot was performed for antisera obtained from mice immunized with: (**A**) PBS, (**B**) *E. coli* DH5α, (**C**) EJK001 (IB5230-ch1-kan), (**D**) EJK002 (IB5230-ch1-kan-*toxT*-139F), (**E**) O395, and (**F**) YJB001 (O395-*toxT*-139F). Also shown are results for (**G**) anti-TcpA and (**H**) Coomassie Brilliant Blue stained SDS-PAGE images of purified TcpA-ET and TcpA-cla. Lane E: purified His-tagged TcpA-ET, lane C: purified His-tagged TcpAcla. Arrow indicates His-tagged TcpA (approximately 24 kDa).

As the antibody that was raised against an internal fragment of classical TcpA reacted with TcpA-cla, TcpA-ET, and Haitian type TcpA by western blot (Figs. 1 and 2, and S4), antiserum that was obtained using the classical biotype strain also reacted with TcpA-cla, TcpA-ET, and Haitian type TcpA (Figs. 2C, 2D, and 3). Antiserum that was raised against the Wave 3 strain expressing Haitian TcpA (EJK002) also reacted with Haitian type TcpA, TcpA-ET, and TcpA-cla (Figs. 2B and 3).

To confirm the recognition of cellular TcpA by the antiserum, purified TcpA-ET and TcpA-cla (Fig. S2) were analyzed by western blot (Fig. 3). Purified TcpA-ET and TcpA-cla were recognized by antiserum that was raised against TcpA-expressing EJK002, O395, and YJB001 (Figs. 3D-3F) but not against *E. coli* or EJK001 (Figs. 3B and 3C).

Next, we examined TcpA-specific serum IgG levels in antisera by ELISA. TcpA type-specific antibody titers increased in 2-fold and 16-fold in antisera against EJK002 and YJB001, respectively, when compared with EJK001 (data not shown). These findings validate that robust antibody responses are induced against TcpA by immunization with inactivated *V. cholerae* strains containing the *toxT*-139F allele.

### Expression of TcpA in *V. cholerae* Strains That Are Components of Cholera Vaccines

**Fig. 4:**
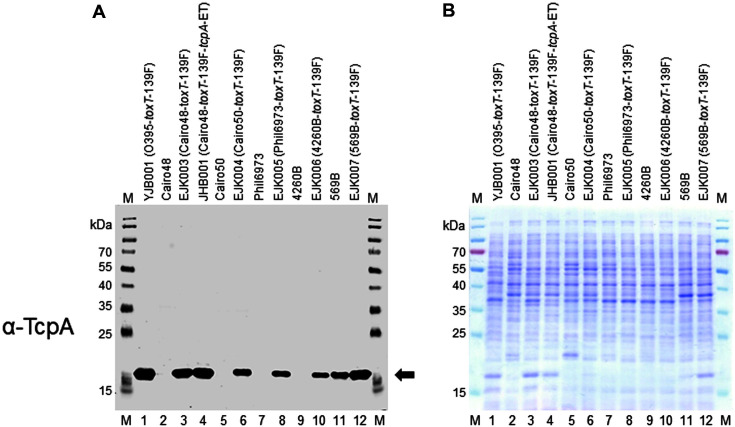
Expression of TcpA in *V. cholerae* strains in oral cholera vaccines. Bacterial strains were cultured at 30°C in LB media except for Cairo50 and EJK004 (Cairo50-*toxT*-139F), which were cultured in AKI media at 30°C (without 4 h of static incubation). (**A**) Cellular TcpA was detected by western blot with anti-TcpA. Approximately 1 × 10^7^ cells were analyzed. M: molecular weight marker (kDa), lane 1: YJB001 (O395-*toxT*-139F), lane 2: Cairo48, lane 3: EJK003 (Cairo48-*toxT*-139F), lane 4: JHB001 (Cairo48-*toxT*-139F-*tcpA*-ET), lane 5: Cairo50, lane 6: EJK004 (Cairo50-*toxT*-139F), lane 7: Phil6973, lane 8: EJK005 (Phil6973-*toxT*-139F), lane 9: 4260B, lane 10: EJK006 (4260B-*toxT*-139F), lane 11: 569B, and lane 12: EJK007 (569B*toxT*-139F). Arrow indicates cellular TcpA. (**B**) Coomassie Brilliant Blue-stained SDS-PAGE of the same bacterial cells.

Because TCP that is expressed via the *toxT*-139F allele in *V. cholerae* strains induced immune responses against TCP, we reasoned that this allele could be introduced to the *V. cholerae* strains in OCVs. Two serogroup O1 classical biotype strains—Cairo48 and Cairo50, the Wave 1 El Tor biotype strain Phil6973, and the O139 serogroup strain 4260B are constituents of OCVs (Table 1). Shanchol and Euvichol contain these 4 strains, whereas Dukoral omits 4260B [16]. These strains contain the *toxT*-139Y allele, and no TCP was expressed by these strains under various culture conditions (LB and AKI media, pH adjustment, and temperature 30°C or 37°C, Fig. 4). In particular, the 2 classical biotype strains Cairo48 and Cairo50 did not express TCP even under agglutinating conditions (data not shown).

**Classical biotype strains Cairo48 and Cairo50.** The *toxT*-139F allele was introduced to Cairo48 and Cairo50 to construct EJK003 (Cairo48-*toxT*-139F) and EJK004 (Cairo50-*toxT*-139F), respectively. Introduction of *toxT*-139F allele facilitated TcpA expression and substantially more TcpA was expressed in EJK003 than EJK004 (lanes 2–6, Fig. 4).

We also introduced the *toxT*-139F allele to another classical biotype strain, 569B, which has been used in a live attenuated OCV [27], to generate the strain EJK007. 569B cells produced TcpA when cultured in LB media at 30°C, and the expression of TcpA was upregulated by the *toxT*-139F allele (lanes 11 and 12, Fig. 4) —properties that were similar to those of O395. However, 569B and EJK007 were not applied in animal experiments in the present study, because the strain 569B is used for the live attenuated OCV. The potential for the *ctxA*-deleted strain of EJK007 to induce immune responses against TcpA as a live-attenuated form remains to be examined.

**El Tor Biotype Strain Phil6973 and O139 Serogroup Strain 4260B**. Phil6973 and 4260B did not express TcpA under various laboratory culture conditions (LB or AKI media, temperature 30°C or 37°C; lanes 7 and 9, Fig. 4). We examined the expression of TcpA in El Tor biotype and O139 serogroup strains under AKI conditions (4 h of static incubation followed by 16 h of shaking culture in AKI media) and observed that the expression of TcpA in these strains was negligible (Fig. S5). Replacement of the *toxT*-139Y allele with *toxT*-139F in these strains (EJK005 and EJK006, respectively) stimulated the TcpA production in LB media at 30°C, albeit at lower levels than EJK003 (lanes 8, 10, and 3 Fig. 4B).

### Construction of a Cairo48 Variant that Expresses El Tor Type TcpA

The expression of TcpA-ET in EJK005 (Phil6973-*toxT*-139F) and EJK006 (4260B-*toxT*-139F) was not as high as the expression of TcpA-cla in EJK003 (Cairo48-*toxT*-139F), which made us postulated that their antibody responses against TcpA-ET were not as robust as those of classical biotype strains. Thus, *tcpA*-cla in EJK003 (Cairo48-*toxT*-139F) was replaced with *tcpA*-ET of El Tor biotype strain N16961 to construct the strain JHB001 (Cairo48-*toxT*-139F-*tcpA*-ET); consequently, the expression of TcpA-ET in this strain was 3.3-fold and 6-fold higher than that of EJK005 and EJK006, respectively (lane 4, 8, 10, Figs. 4A and 4B). Then, JHB001 was also examined for its antibody responses against TcpA-ET in an animal model (described below).

### Antibody Responses against Inactivated OCV Strains

Nine groups of mice were immunized with inactivated bacterial cells as forementioned [PBS as a negative control, *E. coli* DH5α, Cairo48, EJK003 (Cairo48-*toxT*-139F), EJK004 (Cairo50-*toxT*-139F), Phil6973, EJK005 (Phil6973-*toxT*-139F), EJK006 (4260B-*toxT*-139F), and JHB001 (Cairo48-*toxT*-139F-*tcpA*-ET)]. Among *V. cholerae* strains, Cairo48 and Phil6973 do not express TcpA, whereas EJK003, EJK004, EJK005, EJK006, and JHB001 do under simple single-phase culture conditions (Fig. 4). Two other *V. cholerae* strains—Cairo50 and 4260B—which do not express TcpA, were not examined in animal experiments. Anti-TcpA levels in the antisera after the third immunization were analyzed with western blot and ELISA by using His-tagged TcpA-ET and TcpA-cla.

**Fig. 5:**
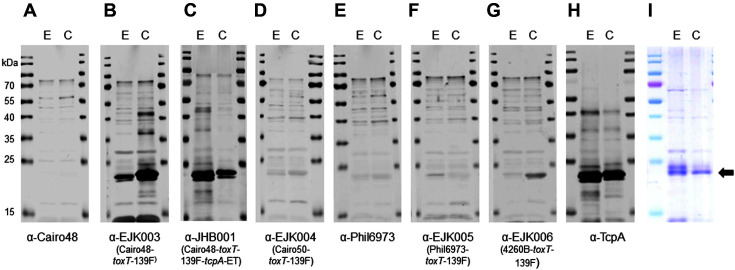
Western blot analysis of His-tagged TcpA-ET and TcpA-cla with mouse antisera raised by inactivated bacterial strains. Western blot was performed antisera obtained from mice immunized with (**A**) Cairo48, (**B**) EJK003 (Cairo48-*toxT*-139F), (**C**) JHB001 (Cairo48-*toxT*-139F-*tcpA*-ET), (**D**) EJK004 (Cairo50-*toxT*-139F), (**E**) Phil6973, (**F**) EJK005 (Phil6973-*toxT*-139F), (**G**) EJK006 (4260B-*toxT*-139F). Also shown are results for (**H**) anti-TcpA and (**I**) Coomassie Brilliant Blue stained SDS-PAGE images of purified TcpA-ET and TcpA-cla. Lane E: purified His-tagged TcpA-ET, lane C: purified Histagged TcpA-cla. Arrow indicates His-tagged TcpA (approximately 24 kDa).

The results showed that mouse antiserum against *V. cholerae* strains that do not express TcpA (Cairo48 and Phil6973) did not react with purified TcpA (Figs. 5A and 5E). Inactivated *V. cholerae* strains that expressed TcpA via the *toxT*-139F allele (EJK003, EJK004, EJK005, EJK006, and JHB001) induced antibody responses against TcpA in immunized mice (Figs. 5B-D, D, 5F, and 5G). Notably, the level of TcpA expression correlated with the intensity of antibody responses against TcpA. EJK003 expressed higher amounts of TcpA than other strains and antiserum against EJK003 reacted with TcpA more intensely than those against EJK004, EJK005, and EJK006.

Despite the cross-reactivity among TcpA-ET and TcpA-cla, the recognition of TcpA-ET by sera against EJK005 and EJK006, which express TcpA-ET, was not as robust as that against EJK003, which expresses TcpA-cla (Figs. 5B, 5F, and 5G). Thus, we sought to induce strong antibody responses against TcpA-ET by JHB001 (Cairo48-*toxT*-139F-*tcpA*-ET). As anticipated, antiserum against JHB001 reacted intensely with TcpA-ET (Fig. 5C). Moreover, antiserum against TcpA-cla reacted with TcpA-cla more intensely than TcpA-ET, and antiserum that was obtained with TcpA-ET recognized TcpA-ET better than TcpA-cla (Figs. 5B and 5C). The recognition of TcpA by sera was confirmed by ELISA, which demonstrated 8-fold and 4-fold increases in TcpA-cla and TcpA-ET type-specific antibody titers in antisera against EJK003 and JHB001, respectively, compared with that against Cairo48.

## Discussion

Despite the World Health Organization campaign ‘Ending Cholera-A Global Roadmap to 2030’, cholera remains a global health problem [39]. Appropriate water supplies and hygiene could prevent cholera the most in endemic areas. In addition, OCVs are being suggested to control cholera [40]. However, the limited protective efficacy of OCVs, poor immune responses in children together with an importunate cost for a multiple-vaccination regimen still need to be addressed [40, 41].

Analyses of immune responses following *V. cholerae* infection have demonstrated antibody reactions against O-antigens, CT, TCP, and other antigens are critical in protective immunity against *V. cholerae* [18, 19]. Among them, such responses against O-antigens and CT (especially CTB) highly correlate with protective efficacy [19, 22, 26]. Whether antibody responses against TCP are important in protection against subsequent *V. cholerae* infection remain to be more clearly determined.

Nevertheless, various attempts have been made to incorporate TCP into cholera vaccines as expressed pilus in bacteria or subunit vaccines [28, 29, 42, 43]. Purified recombinant TcpA expressed in *E. coli* and synthetic internal peptides of TcpA induce robust antibody responses and protective immunity in animal models [28, 30]. *V. cholerae* strains that have been genetically manipulated to produce TCP through a rhamnose-inducible promoter have been developed, but their ability to elicit antibody responses against TCP in an animal model has not been examined [42]. The expression of TcpA in other organisms, such as yeast and tomato, has been examined for potential subunit vaccines [44]. However, more studies are required to determine the clinical value of TcpA in cholera vaccines in humans.

Various culture conditions have been applied for inducing TCP/CT in *V. cholerae* strains [32, 33, 35]. We have demonstrated that the production of CT and TCP in *V. cholerae* strains under simple single-phase laboratory culture conditions can be elicited by the *toxT*-139F allele [37, 38]. Because *V. cholerae* strains that harbor the *toxT*-139F allele can be readily transduced by CTXΦ [38], we expected that such strains likely express functional TCP and can thus be used in vaccines to induce antibody responses against TcpA.

In this study, we found that *V. cholerae* strains that bore the *toxT*-139F allele stably expressed TcpA under simple single-phase laboratory culture conditions and induced antibody responses toward TcpA in an animal model. TcpA-ET and TcpA-cla have 85% amino acid identity, and we observed that antisera that were raised against each type of TcpA cross-reacted with each other. Cross-reactivity between TcpA-cla and TcpA-ET, however, does not assure the cross-protective immunity, an aspect that remains to be examined.

We have previously reported that the *toxT*-139F allele induce CT production in *V. cholerae* strains that are grown under laboratory conditions; however, CT is secreted during culture without any stimulus [37]. When we examined antibody responses against CT in antisera against *V. cholerae* strains that harbored the *toxT*-139F allele, we did not observe any CT-specific antibody responses (data not shown).

We see the limitation of this study that the inactivated bacteria delivered intraperitoneally caused serum IgG responses to TcpA. Although OCVs are recommended for preventing cholera and although mucosal antibody reactions are critical to *V. cholerae* infection, there has been no appropriate animal model for evaluating the efficacy of OCVs [45].

To compensate for those limitations, we developed TCP-expressing *V. cholerae* strains that have been applied to inactivated OCVs. Because antibody responses against O-antigens were already observed in these vaccine strains, we hypothesized that additional antibody responses against TCP could be elicited by incorporating the *toxT*-139F allele into these strains. Immune responses against TCP and protective efficacies of the *V. cholerae* strains developed in this study upon oral delivery in an animal model are being investigated.

Inactivated OCVs used 3 or 4 *V. cholerae* strains, of which Cairo48 could be replaced by EJK003 (Cairo48-*toxT*-139F) or JHB001 (Cairo48-*toxT*-139F-*tcpA*-ET) to induce immune responses against TcpA-cla and TcpA-ET, respectively. Moreover, the *toxT*-139F allele could also be introduced into live-attenuated OCVs. We have demonstrated the expression of TcpA through the addition of the *toxT*-139F allele to the classical biotype strain 569B, which has been used in live-attenuated OCVs.

Our results demonstrate that *V. cholerae* strains that harbor the *toxT*-139F allele induce antibody responses against TCP, highlighting their significant potential as cholera vaccines.

## Supplemental Materials

Supplementary data for this paper are available on-line only at http://jmb.or.kr.

## Figures and Tables

**Table 1:** *V. cholerae* strains used in this study.

Strains	Note	Genome sequence information and reference
O395	Classical biotype strain, *toxT*-139Y	CP000626/CP000627 [4]
YJB001 (O395-*toxT*-139F)	*toxT*-139F	[37]
N16961	Wave 1 El Tor biotype strain, *toxT*-139Y	AE003852/AE003853 [46]
YJB003 (N16961-*toxT*-139F)	*toxT*-139F	[37]
T19479	Wave 1 El Tor biotype strain, *toxT*-139Y	ERS013250 [4]
YJB006 (T19479-*toxT*-139F)	*toxT*-139F	[37]
IB5230	Wave 3 El Tor variant (2010 Haitian cholera outbreak strain), *toxT*-139Y	AELH00000000.1[13]
EJK001 (IB5230-ch1-kan)	*ctxAB* of IB5230 was replaced by a kanamycin resistance cassette	This study
EJK002 (IB5230-ch1-kan-*toxT*-139F)	*ctxAB* of IB5230 was replaced by a kanamycin resistance cassette , *toxT*-139F	This study
Cairo48	Classical biotype strain, *toxT*-139Y	ERS013171 nature
EJK003 (Cairo48-*toxT*-139F)	*toxT*-139F	This study
JHB001 (Cairo48-*toxT*-139F-*tcpA*-ET)	*tcpA*-cla was replaced by *tcpA*-ET	This study
Cairo50	Classical biotype strain, *toxT*-139Y	ERS013165 nature
EJK004 (Cairo50-*toxT*-139F)	*toxT*-139F	This study
Phil6973	Wave 1 El Tor biotype strain, *toxT*-139Y	ERS013248 nature
EJK005 (Phil6973-*toxT*-139F)	*toxT*-139F	This study
4260B	Serogroup O139 strain, *toxT*-139Y	AMVL01000000
EJK006 (4260B-*toxT*-139F)	*toxT*-139F	This study
569B	Classical biotype strain, *toxT*-139Y	DADXPZ010000000
EJK007 (569B-*toxT*-139F)	*toxT*-139F	This study
V212-1	Wave 2 El Tor variant	ERS013132 [4]
MG116025	Wave 2 El Tor variant , intrinsic *toxT*-139F	ERS013135 [4]

## References

[1] Clemens JD, Nair GB, Ahmed T, Qadri F, Holmgren J (2017). Cholera. Lancet.

[2] Koch R (1884). An address on cholera and its Bacillus. Br. Med. J..

[3] Kaper JB, Morris JG, Levine MM (1995). Cholera. Clin. Microbiol. Rev..

[4] Mutreja A, Kim DW, Thomson NR, Connor TR, Lee JH, Kariuki S (2011). Evidence for several waves of global transmission in the seventh cholera pandemic. Nature.

[5] Safa A, Nair GB, Kong RY (2010). Evolution of new variants of *Vibrio cholerae* O1. Trends Microbiol..

[6] Kim EJ, Lee CH, Nair GB, Kim DW (2015). Whole-genome sequence comparisons reveal the evolution of *Vibrio cholerae* O1. Trends Microbiol..

[7] Herrington DA, Hall RH, Losonsky G, Mekalanos JJ, Taylor RK, Levine MM (1988). Toxin, toxin-coregulated pili, and the toxR regulon are essential for *Vibrio cholerae* pathogenesis in humans. J. Exp. Med..

[8] Lim MS, Ng D, Zong Z, Arvai AS, Taylor RK, Tainer JA (2010). *Vibrio cholerae* El Tor TcpA crystal structure and mechanism for pilus-mediated microcolony formation. Mol. Microbiol..

[9] Krebs SJ, Taylor RK (2011). Protection and attachment of *Vibrio cholerae* mediated by the toxin-coregulated pilus in the infant mouse model. J. Bacteriol..

[10] Waldor MK, Mekalanos JJ (1996). Lysogenic conversion by a filamentous phage encoding cholera toxin. Science.

[11] Li J, Lim MS, Li S, Brock M, Pique ME, Woods VL (2008). *Vibrio cholerae* toxin-coregulated pilus structure analyzed by hydrogen/deuterium exchange mass spectrometry. Structure.

[12] Iredell JR, Manning PA (1994). Biotype-specific *tcpA* genes in *Vibrio cholerae*. FEMS Microbiol. Lett..

[13] Chin CS, Sorenson J, Harris JB, Robins WP, Charles RC, Jean-Charles RR (2011). The origin of the Haitian cholera outbreak strain. N. Engl. J. Med..

[14] Ghosh P, Naha A, Basak S, Ghosh S, Ramamurthy T, Koley H (2014). Haitian variant *tcpA* in *Vibrio cholerae* O1 El Tor strains in Kolkata, India. J. Clin. Microbiol..

[15] Cheney L, Payne M, Kaur S, Lan R (2021). Multilevel genome typing describes short- and long-term *Vibrio cholerae* molecular epidemiology. mSystems.

[16] Shaikh H, Lynch J, Kim J, Excler JL (2020). Current and future cholera vaccines. Vaccine.

[17] Ali M, Emch M, Park JK, Yunus M, Clemens J (2011). Natural cholera infection-derived immunity in an endemic setting. J. Infect. Dis..

[18] Harris AM, Bhuiyan MS, Chowdhury F, Khan AI, Hossain A, Kendall EA (2009). Antigen-specific memory B-cell responses to *Vibrio cholerae* O1 infection in Bangladesh. Infect. Immun..

[19] Charles RC, Nakajima R, Liang L, Jasinskas A, Berger A, Leung DT (2017). Plasma and mucosal immunoglobulin M, immunoglobulin A, and immunoglobulin G responses to the *Vibrio cholerae* O1 protein immunome in adults with cholera in Bangladesh. J. Infect. Dis..

[20] Kauffman RC, Bhuiyan TR, Nakajima R, Mayo-Smith LM, Rashu R, Hoq MR (2016). Single-cell analysis of the plasmablast response to *Vibrio cholerae* demonstrates expansion of cross-reactive memory B cells. mBio.

[21] Lopez AL, Gonzales ML, Aldaba JG, Nair GB (2014). Killed oral cholera vaccines: history, development and implementation challenges. Ther. Adv. Vaccines..

[22] Holmgren J (2021). An update on cholera immunity and current and future cholera vaccines. Trop. Med. Infect. Dis..

[23] McCarty JM, Lock MD, Hunt KM, Simon JK, Gurwith M (2018). Safety and immunogenicity of single-dose live oral cholera vaccine strain CVD 103-HgR in healthy adults age 18-45. Vaccine.

[24] Levine MM, Kaper JB, Herrington D, Ketley J, Losonsky G, Tacket CO (1988). Safety, immunogenicity, and efficacy of recombinant live oral cholera vaccines, CVD 103 and CVD 103-HgR. Lancet.

[25] Aktar A, Rahman MA, Afrin S, Akter A, Uddin T, Yasmin T (2018). Plasma and memory B cell responses targeting O-specific polysaccharide (OSP) are associated with protection against *Vibrio cholerae* O1 infection among household contacts of cholera patients in Bangladesh. PLoS Negl. Trop. Dis..

[26] Islam K, Hossain M, Kelly M, Mayo Smith LM, Charles RC, Bhuiyan TR (2018). Anti-O-specific polysaccharide (OSP) immune responses following vaccination with oral cholera vaccine CVD 103-HgR correlate with protection against cholera after infection with wild-type *Vibrio cholerae* O1 El Tor Inaba in North American volunteers. PLoS Negl. Trop. Dis..

[27] Mayo-Smith LM, Simon JK, Chen WH, Haney D, Lock M, Lyon CE (2017). The live attenuated cholera vaccine CVD 103-HgR primes responses to the toxin-coregulated pilus antigen TcpA in subjects challenged with wild-type *Vibrio cholerae*. Clin. Vaccine Immunol..

[28] Rollenhagen JE, Kalsy A, Cerda F, John M, Harris JB, Larocque RC (2006). Transcutaneous immunization with toxincoregulated pilin A induces protective immunity against *Vibrio cholerae* O1 El Tor challenge in mice. Infect. Immun..

[29] Asaduzzaman M, Ryan ET, John M, Hang L, Khan AI, Faruque ASG (2004). The major subunit of the toxin-coregulated pilus TcpA induces mucosal and systemic immunoglobulin A immune responses in patients with cholera caused by *Vibrio cholerae* O1 and O139. Infect. Immun..

[30] Taylor RK, Kirn TJ, Meeks MD, Wade TK, Wade WF (2004). A *Vibrio cholerae* classical TcpA amino acid sequence induces protective antibody that binds an area hypothesized to be important for toxin-coregulated pilus structure. Infect. Immun..

[31] Yu RR, DiRita VJ (2002). Regulation of gene expression in *Vibrio cholerae* by ToxT involves both antirepression and RNA polymerase stimulation. Mol. Microbiol..

[32] Sanchez J, Medina G, Buhse T, Holmgren J, Soberon-Chavez G (2004). Expression of cholera toxin under non-AKI conditions in *Vibrio cholerae* El Tor induced by increasing the exposed surface of cultures. J. Bacteriol..

[33] Cobaxin M, Martinez H, Ayala G, Holmgren J, Sjoling A, Sanchez J (2014). Cholera toxin expression by El Tor *Vibrio cholerae* in shallow culture growth conditions. Microb. Pathog..

[34] Jonson G, Svennerholm A-M, Holmgren J (1990). Expression of virulence factors by classical and El Tor *Vibrio cholerae*
*in vivo* and *in vitro*. FEMS Microbiol. Lett..

[35] Iwanaga M, Yamamoto K, Higa N, Ichinose Y, Nakasone N, Tanabe M (1986). Culture conditions for stimulating cholera toxin production by *Vibrio cholerae* O1 El Tor. Microbiol. Immunol..

[36] Lee D, Kim EJ, Baek Y, Lee J, Yoon Y, Nair GB (2020). Alterations in glucose metabolism in *Vibrio cholerae* serogroup O1 El Tor biotype strains. Sci. Rep..

[37] Baek Y, Lee D, Lee J, Yoon Y, Nair GB, Kim DW (2020). Cholera toxin production in *Vibrio cholerae* O1 El Tor biotype strains in single-phase culture. Front. Microbiol..

[38] Kim EJ, Yu HJ, Lee JH, Kim JO, Han SH, Yun CH (2017). Replication of *Vibrio cholerae* classical CTX phage. Proc. Natl. Acad. Sci. USA.

[39] Legros D, Partners of the Global Task Force on Cholera C (2018). Global cholera epidemiology: Opportunities to reduce the burden of cholera by 2030. J. Infect. Dis..

[40] Harris JB (2018). Cholera: Immunity and prospects in vaccine development. J. Infect. Dis..

[41] Teoh SL, Kotirum S, Hutubessy RCW, Chaiyakunapruk N (2018). Global economic evaluation of oral cholera vaccine: A systematic review. Hum. vaccin. Immunother..

[42] Hauke CA, Taylor RK (2017). Production of putative enhanced oral cholera vaccine strains that express toxin-coregulated pilus. PLoS One.

[43] Zareitaher T, Sadat Ahmadi T, Latif Mousavi Gargari S (2022). Immunogenic efficacy of DNA and protein-based vaccine from a chimeric gene consisting OmpW, TcpA and CtxB of *Vibrio cholerae*. Immunobiology.

[44] Sharma MK, Singh NK, Jani D, Sisodia R, Thungapathra M, Gautam JK (2008). Expression of toxin co-regulated pilus subunit A (TcpA) of *Vibrio cholerae* and its immunogenic epitopes fused to cholera toxin B subunit in transgenic tomato (*Solanum lycopersicum*). Plant Cell Rep..

[45] Sit B, Fakoya B, Waldor MK (2022). Animal models for dissecting *Vibrio cholerae* intestinal pathogenesis and immunity. Curr. Opin. Microbiol..

[46] Heidelberg JF, Eisen JA, Nelson WC, Clayton RA, Gwinn ML, Dodson RJ (2000). DNA sequence of both chromosomes of the cholera pathogen *Vibrio cholerae*. Nature.

