# Supplementary material for: Inactivated Vibrio cholerae Strains That Express TcpA via the toxT-139F Allele Induce Antibody Responses against TcpA
Source: J Microbiol Biotechnol. 2022 Oct 12;32(11):1396–405. doi: 10.4014/jmb.2209.09001 (PMC9720071; doi:10.4014/jmb.2209.09001)
Supplement: Supplementary file 1 [file jmb-32-11-1396-supple.pdf]

J. Microbiol. Biotechnol.
https://doi.org/10.4014/jmb.2209.09001

Supple. Fig. S1.

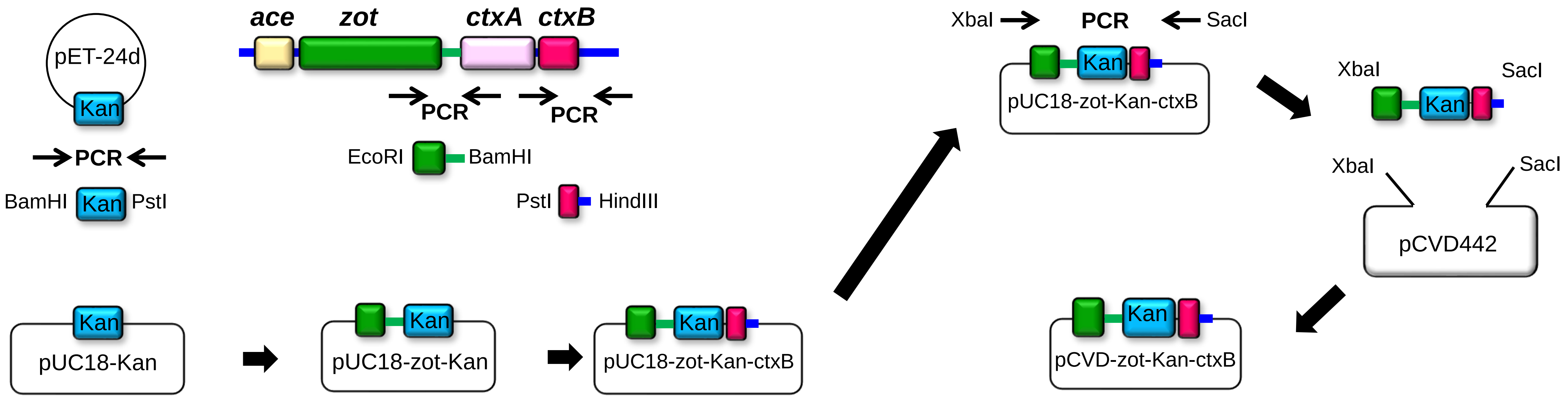

Fig. S1. Construction of pCVD-zot-Kan-ctxB suicide plasmid. Details are described in the Materials and Methods. *E. coli* strain SM10 was transformed with pCVD-zot-Kan-ctxB and the entire *ctxA* and the first 166 bp of *ctxB* of strain IB5230 was replaced with a kanamycin resistance cassette by allelic exchange method to generate strain EJK001 (IB5230-ch1-kan).

Supple. Fig. S2.

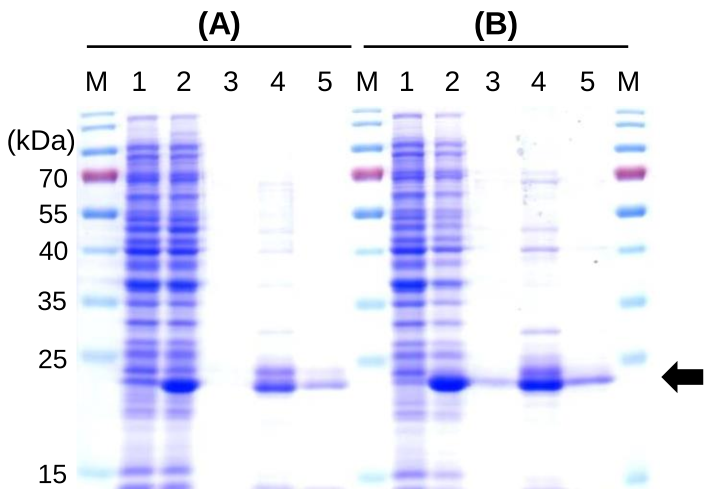

Fig. S2. Purification of His-tagged TcpA-ET and TcpA-cla.

E. coli BL21(DE3) strains harboring pET24-TcpA-ET and pET24-TcpA-Cla were cultured in LB media. Expression of TcpA-El Tor (A) and TcpA-cla (B) was induced by adding 1 mM IPTG. The cells were harvested and disrupted by freeze-thaw method and sonication. The cytoplasmic fraction was obtained by centrifugation of the disrupted cells and loaded onto TALON resin. Eluted fractions were analyzed by SDS-PAGE. Lane 1: *E. coli* BL21 (DE3) without IPTG induction as a negative control, lane 2: induction of TcpA expression in *E. coli* BL21 (DE3) by treatment with 1 mM IPTG for 3 h, lane 3 - 5: eluted fractions. Arrow indicates His-tagged TcpA with a molecular weight of approximately 24 kDa.

Supple. Fig. S3.

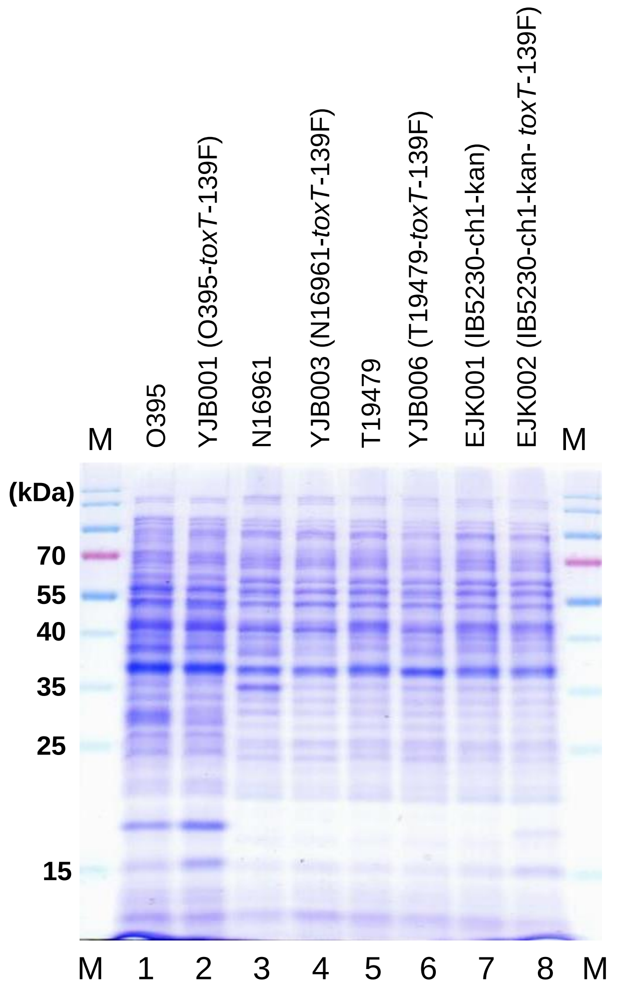

Fig. S3. Coomassie Brilliant Blue-stained SDS-PAGE gel image of *V. cholerae* strains. M: molecular weight marker (kDa), lane 1: O395, lane 2: YJB001 (O395-*toxT*-139F), lane 3: N16961, lane 4: YJB003 (N16961-*toxT*-139F), lane 5: T19479, lane 6: YJB006 (T19479-*toxT*-139F), lane 7: EJK001 (IB5230-ch1-kan), and lane 8: EJK002 (IB5230-ch1-kan- *toxT*-139F).

Supple. Fig. S4.

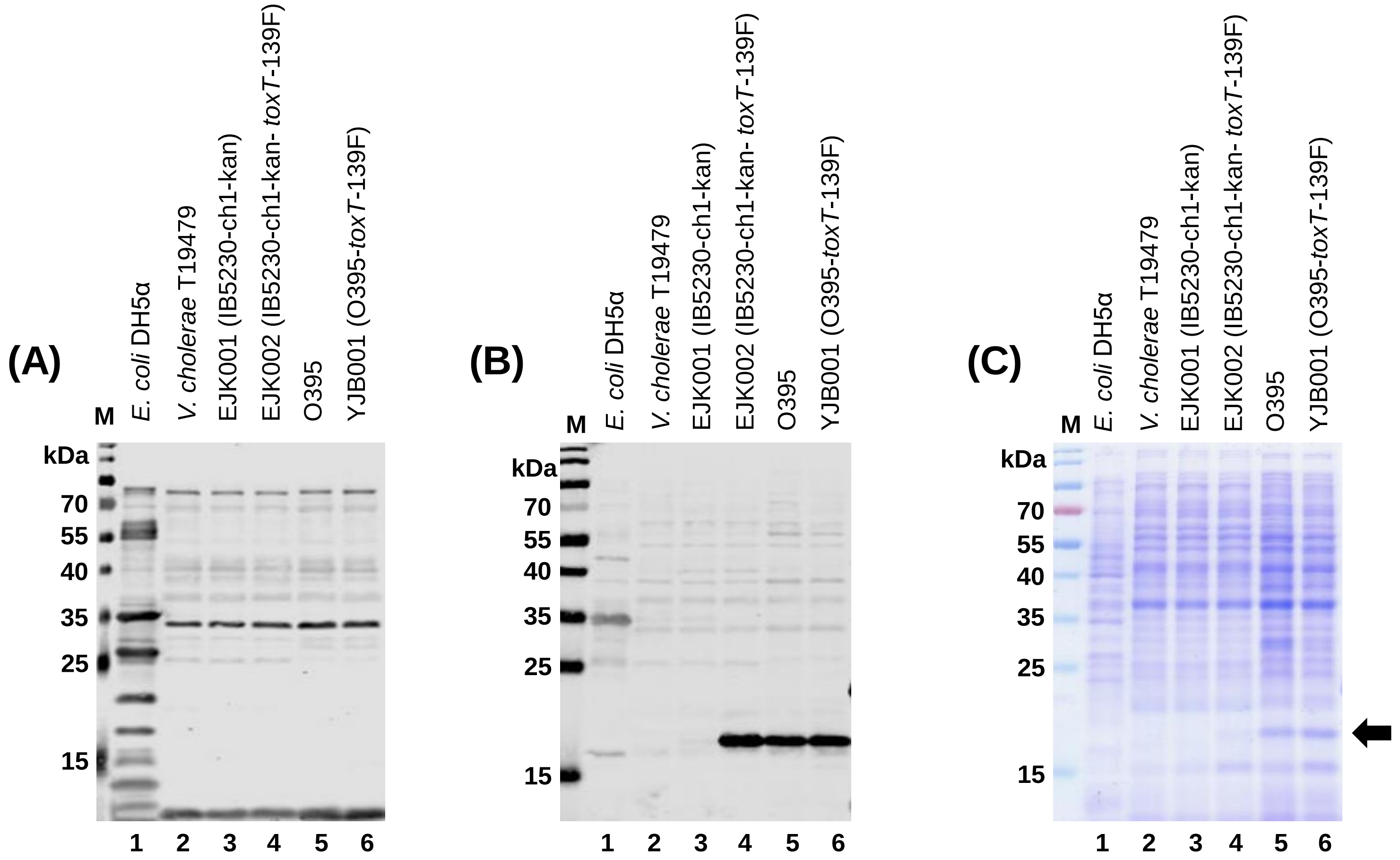

Fig. S4. Western blot analysis of bacterial strains using mouse antiserum in response to *E. coli* and anti-TcpA. (A) Western blot with antisera raised against *E. coli* DH5α, (B) western blot with anti-TcpA antibody, and (C) Coomassie Brilliant Blue-stained SDS-PAGE gel of the same samples. Approximately 10^7 bacterial cells were analyzed. Lane 1: *E. coli* DH5α, lane 2: *V. cholerae* T19479, lane 3: EJK001 (IB5230-ch1-kan), lane 4: EJK002 (IB5230-ch1-kan- *toxT*-139F), lane 5: O395, and lane 6: YJB001 (O395-*toxT*-139F). Arrow indicates cellular TcpA (approximately 21 kDa).

Supple. Fig. S5.

Lane 1 LB: 30°C LB
Lane 2, 4, 6, 8, 10 LB: 37°C LB
AKI: 37°C AKI, static incubation 4 hr + shaking culture 16 hr

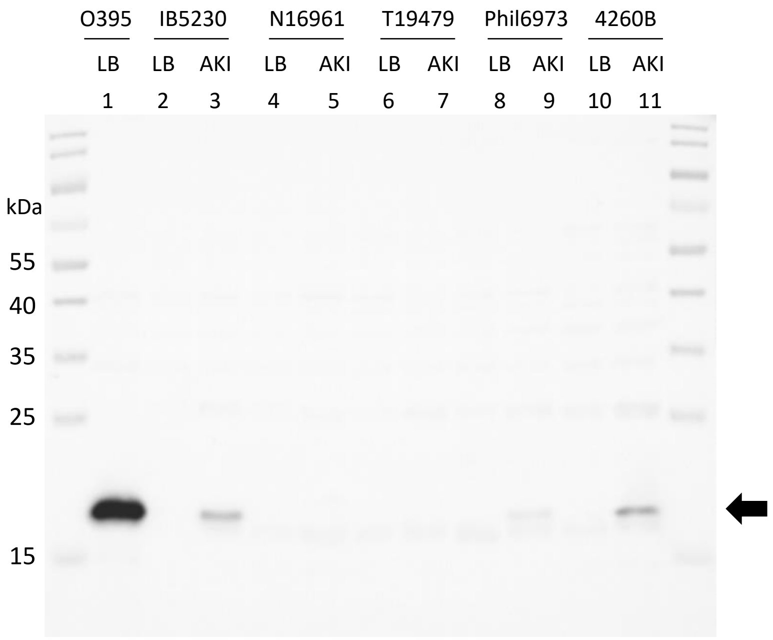

α-TcpA

Fig. S5. TcpA expression in El Tor biotype strains (IB5230, N16961, T19479, and Phil6973) and O139 serogroup strain (4260B).
Bacterial strains were cultured at in LB media at 30℃ (O395; lane 1), or at 37℃ (lane 2: IB5230, lane 4: N16961, Lane 6: T19479, lane 8: Phil6973, and lane 10: 4260B). The same bacterial strains were also cultured under AKI conditions (4 h of static incubation followed by 16 h of shaking culture in AKI media at 37℃). Lane 3: IB5230, lane 5: N16961, lane 7: T19479, Lane 9: Phil6973, and lane 11: 4260B). TcpA was detected by western blot with anti-TcpA. Arrow indicates cellular TcpA (approximately 21 kDa).
